# Local shape volume alterations in subcortical structures of suicide attempters with major depressive disorder

**DOI:** 10.1002/hbm.25168

**Published:** 2020-08-17

**Authors:** Wooyoung Kang, Jeong‐Hyeon Shin, Kyu‐Man Han, Aram Kim, Youbin Kang, June Kang, Woo‐Suk Tae, Jong‐Woo Paik, Hae‐Woo Lee, Joon‐Kyung Seong, Byung‐Joo Ham

**Affiliations:** ^1^ Department of Biomedical Sciences Korea University College of Medicine Seoul Republic of Korea; ^2^ Medical & Health Device Division Korea Testing Laboratory Seoul Republic of Korea; ^3^ Department of Psychiatry Korea University Anam Hospital, Korea University College of Medicine Seoul Republic of Korea; ^4^ Department of Brain and Cognitive Engineering Korea University Seoul Republic of Korea; ^5^ Brain Convergence Research Center Korea University Anam Hospital Seoul Republic of Korea; ^6^ Department of Neuropsychiatry College of Medicine, Kyung Hee University Seoul Republic of Korea; ^7^ Department of Psychiatry Seoul Medical Center Seoul Republic of Korea; ^8^ School of Biomedical Engineering, Department of Artificial Intelligence Korea University Seoul Republic of Korea

**Keywords:** basal ganglia, LCSPT circuits, local shape volume, major depressive disorder, stress, suicidality, thalamus

## Abstract

Suicide is among the most important global health concerns; accordingly, an increasing number of studies have shown the risks for suicide attempt(s) in terms of brain morphometric features and their clinical correlates. However, brain studies addressing suicidal vulnerability have been more focused on demonstrating impairments in cortical structures than in the subcortical structures. Using local shape volumes (LSV) analysis, we investigated subcortical structures with their clinical correlates in depressed patients who attempted suicide. Then we compared them with depressed patients without a suicidal history and age‐ and sex‐matched healthy controls (HCs; i.e., 47 suicide attempters with depression, 47 non‐suicide attempters with depression, and 109 HCs). Significant volumetric differences were found between suicidal and nonsuicidal depressed patients in several vertices: 16 in the left amygdala; 201 in the left hippocampus; 1,057 in the left putamen; and 140 in the left pallidum; 1 in the right pallidum; and 6 in the bilateral thalamus. These findings indicated subcortical alterations in LSV in components of the limbic–cortical–striatal–pallidal–thalamic circuits. Moreover, our results demonstrated that the basal ganglia was correlated with perceived stress levels, and the thalamus was correlated with suicidal ideation. We suggest that suicidality in major depressive disorder may involve subcortical volume alterations.

## Graphical abstract

Using local shape volumes (LSV) analysis, significant volumetric differences were found between suicidal and nonsuicidal depressed patients in the left amygdala, left hippocampus, left putamen, bilateral pallidum, and thalamus. These findings propose that suicidality in major depressive disorder may involve subcortical volumetric alterations.

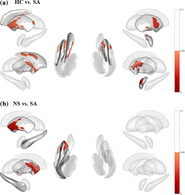

## 1 INTRODUCTION

Suicide is a critical global health concern and is associated with a substantial socioeconomic burden. In fact, about 800,000 individuals die from suicide every year, which corresponds to one individual every 40 seconds (World Health Organization, 2018). In particular, South Korea was ranked the 10th among countries with the highest suicide rates in 2020, and 90% of suicide victims are estimated to have mental health problems (World Population Review, 2020). The risk for suicide is known to be 10–20 times higher in people with mood disorders than in the general population (Angst, Angst, & Stassen, 1999). A psychological autopsy study of suicide victims reported that 94% had a mental disorder, among which depression was the most prevalent, accounting for 51% of the reported mental disorders (Marttunen, Aro, Henriksson, & Lönnqvist, 1991).

Over the past decade, efforts to specify the neurobiological aspects of suicide risk in major depressive disorder (MDD) have been made by employing magnetic resonance imaging (MRI) (Jollant et al., 2018; van Heeringen, Bijttebier, & Godfrin, 2011; Wagner et al., 2011). Previous structural imaging studies have reported abnormalities in cortical and subcortical structures, including the frontal and temporal lobes, the hippocampus (Gosnell et al., 2016), amygdala (Monkul et al., 2007), and basal ganglia (Ahearn et al., 2001; Vang, Ryding, Träskman‐Bendz, van Westen, & Lindström, 2010; Wagner et al., 2011). Moreover, some studies found impaired frontothalamic (Jia et al., 2014) and frontostriatal circuitry (Zhang, Chen, Jia, & Gong, 2014) in suicidal depressed patients. These circuitry approaches demonstrated that the cognitive dysregulation of inhibitory control on emotion derived from the limbic system may be associated with suicidal behavior in depression (Gosnell et al., 2016; Wagner et al., 2012) and other mood disorders (Benedetti et al., 2011; Soloff et al., 2012). These findings also suggest that, among the subcortical regions, the basal ganglia and thalamus may be particularly relevant for suicidality in depression. However, relatively few studies have investigated the volumes of the basal ganglia or thalamus (Nugent, Davis, Zarate Jr., & Drevets, 2013).

While some studies reported significant structural changes in subcortical regions in suicidal depressed patients (Ahearn et al., 2001; Vang et al., 2010), other studies failed to detect such changes (Gifuni et al., 2016; Jollant et al., 2018; Kim et al., 2019; Rentería et al., 2017). A meta‐analysis from the worldwide Enhancing NeuroImaging Genetics through Meta‐Analysis (ENIGMA) MDD working group (Rentería et al., 2017) reported no significant group differences in any of the subcortical volume measures between suicidal and nonsuicidal depression and suggested that many of the previous reports may be false positives. The inconsistent findings regarding subcortical structures with suicidality and the related depressive symptoms may be resulted from small sample sizes and/or the heterogeneity of MDD and suicidality to determine potential interactions between clinical condition, demographic characteristics and brain structural alterations (Aguilar et al., 2003; Campbell & MacQueen, 2006; Schmaal et al., 2016). There is yet no consensus regarding the subcortical structural abnormalities in those with suicidal depression. It is also true that there are not enough studies on the subcortical regions to uncover neuroanatomical correlates of suicidality and its clinical relevance in depression (Rentería et al., 2017). Therefore, further replications of previous findings are needed (Gosnell et al., 2016). Considering its importance and the aforementioned difficulties in studying suicidal behavior in depression, it is necessary to provide more homogenous sample with a larger number, and more sensitive assessments for more reliable and consistent results about suicide risk in patients with depression (Lee & Kim, 2011) so that it can establish to develop effective suicide‐prevention strategies (van Heeringen et al., 2011).

Here, we examined local shape volume (LSV) in the subcortical structures of MDD patients with and without suicide attempts. LSV on the subcortical surface mesh, which has been successfully used in previous studies (Chung et al., 2017; Koo, Shin, Lim, Seong, & Joo, 2017), measures the extent of local atrophy of the subcortical structure. Although there have been few studies investigating subcortical structures and their associations with suicidality (Hwang et al., 2010; Rentería et al., 2017), majority of studies have focused on traditional VBM or volumetric analyses. Because these analyses were not sensitive to subtle changes in subcortical structures (Lu et al., 2016), we predicted that vertex‐based shape analysis could detect focal atrophy of the subcortical structures. We hypothesized that, compared with those considered to have nonsuicidal MDD (NS), those with suicide attempted (SA) MDD would show widespread altered subcortical volumes on LSV, especially in the basal ganglia and thalamus.

## 2 MATERIALS AND METHODS

### 2.1 Subjects

A total of 94 patients with MDD (of whom 47 attempted suicide and 47 did not) were age‐ and sex‐matched with healthy controls (HCs). MDD patients were recruited from the outpatient psychiatry clinic of Korea University Anam Hospital (Seoul, Republic of Korea) from April 2015 to August 2017. The sample was confirmed as MDD by board‐certified psychiatrists (B.‐J. H., K.‐M. H.) using the Structured Clinical Interview from the *Diagnostic and Statistical Manual of Mental Disorders*, *Fourth Edition* Axis I disorders. Basic demographic information, including age, sex, and education level, as well as clinical information, were collected.

Patients with a history of potentially harmful behavior to self in any form, with the intention of ending one's own life, were categorized as SA. History of present illness regarding the suicidal episodes was acquired during the psychiatric interview in the clinic and through review of medical records, such as emergency room visits due to attempted suicide. Accordingly, 47 patients were defined as SA (20 males, 27 females) and 47 were NS (21 males, 26 females). The age of the suicide and non‐suicide attempters both ranged from 19 to 56 years, with an average of 32.28 and 33.21 years, respectively. The severity of depressive symptoms of all participants was assessed using the 17‐item Hamilton Depression Rating Scale (HDRS) (Hamilton, 1960). Self‐reporting questionnaires were used, including the 19‐item Beck Scale for Suicide Ideation (SSI) (Beck, Kovacs, & Weissman, 1979) to measure the current severity of suicidal ideation (i.e., attitudes, plans, and behaviors to commit suicide in the last 2 weeks); the Perceived Stress Scale (PSS) (Cohen, Kamarck, & Mermelstein, 1983) to measure the degree of how often and how much a person felt stressed in the last month; and the Barratt Impulsiveness Scale (BIS) (Patton, Stanford, & Barratt, 1995) for the degree of impulsivity. All clinical scales were assessed at the time of the MRI scanning.

Patients were excluded from the current study based on the following criteria: comorbidity with any other major psychiatric disorder(s); psychotic features such as delusions or hallucinations; history of a serious or uncontrolled medical illness; any primary neurological illness; and any contraindication to MRI scanning, such as having metal implants or claustrophobia.

A total of 109 healthy adults from the community (48 males, 61 females) 19–65 years of age who responded to an advertisement and agreed to voluntarily participate in the study were recruited. They were similarly assessed using a psychiatric exam and confirmed to be free of any current or past psychiatric disorders. The same exclusion criteria also applied to the HC group.

In accordance with the Declaration of Helsinki, a total of 203 participants provided written informed consent before participation in the study. All participants were free to drop out of the study at any stage; however, no participants dropped out of the study. The study protocol was approved by the Institutional Review Board of Korea University Anam Hospital (IRB No: ED15006).

### 2.2 Image acquisition

Three‐dimensional structural MRI scans were acquired using a 3.0 Tesla Siemens Trio whole‐body imaging system (Siemens Medical Systems, Iselin, NJ, USA). A T1‐weighted magnetization‐prepared rapid gradient‐echo was used (repetition time, 1,900 ms; echo time, 2.6 ms; field of view, 220 mm; matrix size, 256 × 256; 176 coronal slices without gap; voxel size, 1 × 1 × 1 mm^3^, flip angle, 16°; and number of excitations, 1).

### 2.3 Image preprocessing and subcortical shape analysis

The T1‐weighted images from each subject were processed to obtain anatomical parcellations of subcortical structures using FreeSurfer software version 5.1.0 (Martinos Center for Biomedical Imaging, http://surfer.nmr.mgh.harvard.edu). The subcortical structures were parceled into the amygdala, caudate nucleus, hippocampus, pallidum (globus pallidus), putamen, and thalamus in both directions. After parcellation, labeled images were converted to the native anatomical spaces of the input MR data. Next, the subcortical surface mesh was extracted from the labeled images of each subject using Laplacian‐based surface modeling system (Cho, Seong, Jeong, Shin,, & Alzheimer's Disease Neuroimaging Initiative, 2012). Surface‐based registration was achieved by adopting a previously described method to provide vertex correspondences for all subcortical surface meshes (Cho et al., 2013). LSV was defined at each vertex on a subcortical mesh using the method described by Shapira, Shamir, and Cohen‐Or (2008) into the problem setting. Based on a simple ray shooting algorithm, this measures the distance from each vertex to the antipodal surface point along the inward‐normal direction. Apparently, this distance measurement reflects local atrophy on the subcortical surface mesh. Each subcortical structure was composed of 2,562 vertices. Further detailed information regarding this method had been described in previous reports (Chung et al., 2017; Kim et al., 2019; Koo et al., 2017; Shapira et al., 2008).

### 2.4 Statistical analysis

Analysis of covariance (ANCOVA) was used to compare LSVs of the three groups, controlling for the effects of age, sex, level of education, and intracranial volume (ICV). Pair‐wise comparisons were performed using ANCOVA and the false discovery rate (FDR) procedure for multiple comparison correction.

Correlation tests were performed between the LSV of each subcortical structure and the SSI and duration of antidepressant treatment, respectively. Because LSVs often do not follow a normal distribution, Spearman's partial correlation was used to control for the effects of age, sex, level of education, and ICV. Unlike in‐group comparisons, cluster‐based statistics were used for multiple comparison correction (Han, Yoo, Seo, Na, & Seong, 2013). Specifically, we identified clusters composed of connected vertices with their correlation coefficients larger or less than a user‐specified threshold such as 0.3 or −0.3, respectively. In order to evaluate the significance level of a cluster for a given threshold, we randomly permuted ordering of the clinical measurements and then constructed a null distribution according to the maximum cluster sizes of every permutation. The *p*‐value of the specific cluster was determined by the relative position of the original ordering of the measurement in the null distribution. This cluster‐based multiple comparison correction was less conservative than other methods, such as Bonferroni correction or FDR procedure.

## 3 RESULTS

### 3.1 Demographic and clinical characteristics of the subjects

Demographic characteristics of each participant, including age, sex, education level, and clinical characteristics, such as the self‐questionnaires (SSI, PSS, and BIS) and history of present illness (the duration of illness and the duration of antidepressant treatment) are summarized in Table 1. Education level was found to be significantly different among groups (*p* < .05); the SA group had a significantly lower education level compared with the NS and HC groups. Particularly, there was no person above the graduate education level in the SA group. There was no significant difference between the NS and HC. Interestingly, the SA group had significantly lower HDRS‐17 levels compared with the NS group (*p* < .001). However, the SA group had a significantly higher degree of suicidal intention (i.e., SSI), longer illness duration, and more depressive episodes than NS patients.

**TABLE 1:** Demographic and clinical characteristics of patients with MDD and HCs

	SA (*n* = 47)	NS (*n* = 47)	HC (*n* = 109)	*p*‐Value (*F*, *χ* ^2^, *t*)
Demographics				
Age, years	32.28 ± 10.86	33.21 ± 9.37	32.01 ± 10.23	.513 (*F* = 0.670)
Sex (female/male)	27/20	26/21	61/48	.977 (*χ* ^2^ = 0.047)
Education level				
Elementary and middle school	4	1	3	.019 (*χ* ^2^ = 11.834)^a^
High school or college/university	43	37	90	.004 (*χ* ^2^ = 11.250)^b^
Graduated	0	9	16	.008 (*χ* ^2^ = 9.632)^c^ .771 (*χ* ^2^ = 0.519)^d^
ICV	1,326,721.05	1,314,244.62	1,327,861.91	.811 (*F* = 0.210)
Clinical scales				
HDRS‐17 score	14.45 ± 5.39	16.74 ± 5.71	1.42 ± 1.83	<.001 (*F* = 320.830)^a^ <.05 (*t* = 2.005)^b^
SSI	19.60 ± 9.70	13.45 ± 10.12	NA	<.05 (*t* = 3.007)
PSS	24.50 ± 8.47	24.04 ± 7.45	NA	.783 (*t* = 0.277)
BIS	55.00 ± 15.50	52.98 ± 11.80	NA	.479 (*t* = 0.711)
Clinical statement				
Illness duration (months)	47.51 ± 50.82	29.02 ± 30.25	NA	<.05 (*t* = 2.143)
Medication duration (months)	5.81 ± 14.49	9.17 ± 20.63	NA	.36 (*t* = 0.914)
Age of first onset	28.55 ± 10.85	31.26 ± 10.95	NA	.991 (*t* = 1.201)
Number of past episodes	2.66 ± 2.416	0.68 ± 0.862	NA	<.001 (*t* = 5.288)
Number of past suicide attempts	1.64 ± 1.607	NA	NA	—
Drug‐treated patients	43	18	NA	—
Medication			—	—
SSRI	8	4	—	—
SNRI	0	1	—	—
NDRI	2	1	—	—
NaSSA	2	0	—	—
SSRE	1	0	—	—
Combination of AD	28	11	—	—
Lithium	0	0	—	—
AED	5	2	—	—
Lithium + AED	0	0	—	—
AED + AED	0	0	—	—
AP	7	6	—	—
Combination of AP	4	0	—	—

*Note:* Data presented as mean ± *SD* or *n*, unless otherwise indicated. Medication: AD, antidepressants; AED, Anti‐epileptic drugs; AP, Antipsychotics; Combination of AD, Combination of two or more types of antidepressants; NaSSA, Noradrenergic and Specific Serotonergic Antidepressant; NDRI, Norepinephrine‐Dopamine Reuptake Inhibitor; SNRI, Serotonin and Norepinephrine Reuptake Inhibitor; SSRI, Selective Serotonin Reuptake Inhibitor.

Abbreviations: BIS, Baratt Impulsiveness Scale; HC, healthy control; HDRS‐17, Hamilton Depression Rating Scale 17; ICV, intracranial volume; MDD, major depressive disorder; NA, not applicable; NS, nonsuicidal MDD; PSS, Perceived Stress Scale; SA, suicide attempted MDD; SSI, Beck Scale for Suicide Ideation.

^a^ Comparisons among the three groups.

^b^ Comparisons between suicidal and nonsuicidal MDD group.

^c^ Comparisons between suicidal MDD and HC.

^d^ Comparisons between nonsuicidal MDD and HC.

### 3.2 Group comparison of LSVs

**FIGURE 1:**
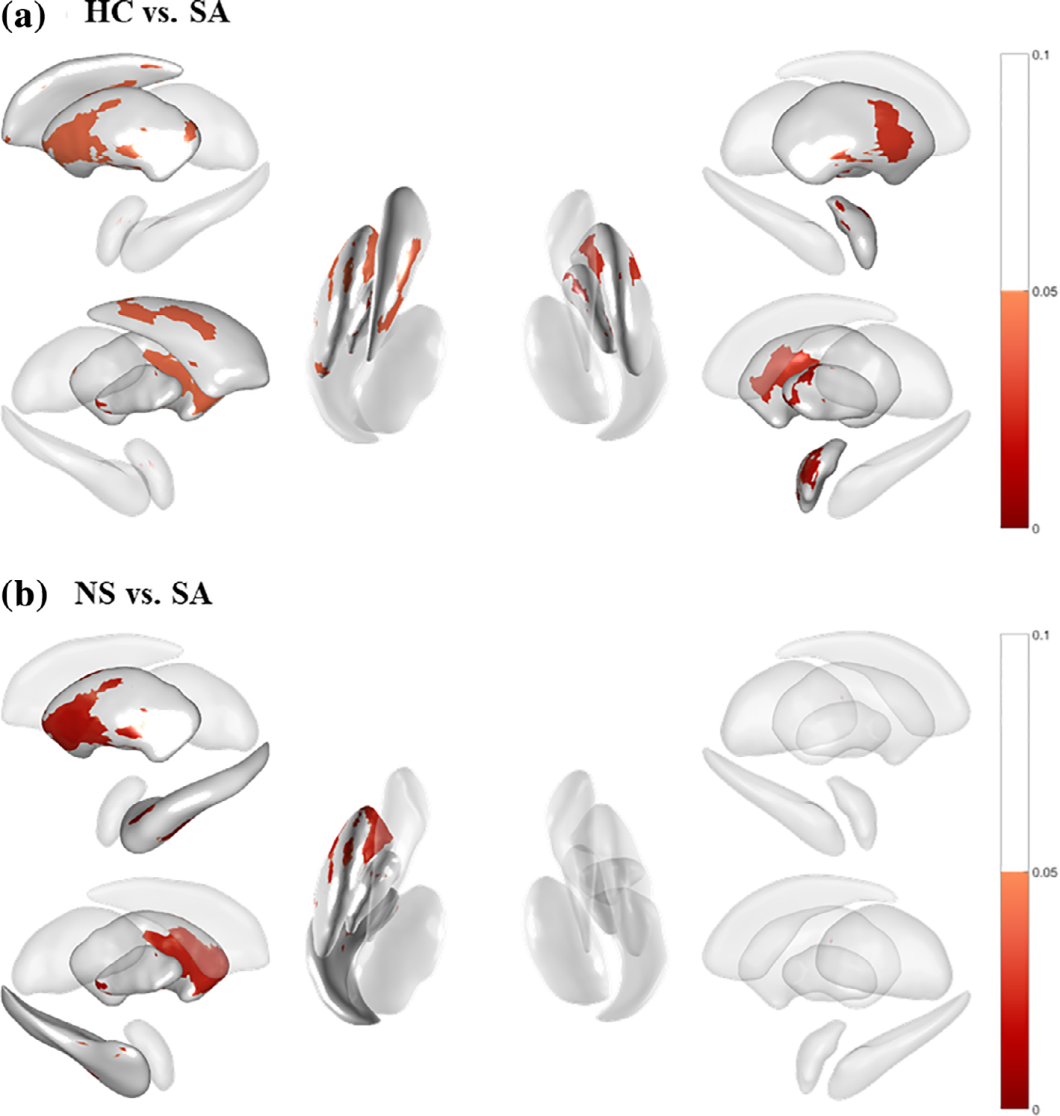
Comparison of subcortical local shape volumes between groups. (a) Shape differences of subcortical structures between HC and SA. (b) Between NS and SA. Color bar shows adjusted *p* value (FDR correction). HC, healthy control; MDD, major depressive disorder; NS, nonsuicidal MDD; SA, suicide attempted MDD

In the left amygdala, left hippocampus, left putamen, and bilateral pallidum and thalamus, significantly localized atrophy was found in the SA group compared to the NS group (Figure 1). Table 2 shows the numbers of vertices in the SA group that were smaller in volume compared to the NS and HC groups. A total of 2,562 vertices showed major LSV differences between the SA and NS groups, with 201; 140; and 1,057 in the left hippocampus, left pallidum, and left putamen, respectively.

**TABLE 2:** Subcortical LSV differences in the number of vertices between groups

Subcortical structure	Three group	SA vs. NS	SA vs. HC	NS vs. HC
	Uncorrected *p*	Corrected *p*	Corrected *p*	Corrected *p*
L amygdala	46	16	25	0
L caudate	379	0	306	0
L hippocampus	335	201	2	0
L pallidum	188	140	160	0
L putamen	1,129	1,057	940	0
L thalamus	7	6	0	6
R amygdala	595	0	474	0
R caudate	74	0	0	8
R hippocampus	257	0	0	206
R pallidum	574	1	550	0
R putamen	470	0	406	0
R thalamus	6	6	0	5

Abbreviations: Corrected *p*, FDR adjusted *p*‐value; HC, healthy control; LSV, local shape volume; MDD, major depressive disorder; NS, nonsuicidal MDD; SA, suicide attempted MDD.

Compared to the HC group, the SA group exhibited significantly reduced LSV in the amygdala, left caudate, globus pallidus, and putamen (Figure 1, Table 2). The NS group exhibited significantly reduced LSV in the right hippocampus. The SA and NS groups significantly differed in reduced LSV in the left hemisphere (Table 2). The HC and NS groups had no significant differences in regions other than the right hippocampus and local regions of other subcortical structures (Table 2).

We conducted a second analysis with SSI score and antidepressant treatment duration as covariates. The left caudate was significantly different in 808 vertices (Supplementary Figure S1).

### 3.3 Correlations between clinical measurement and LSV measures

**FIGURE 2:**
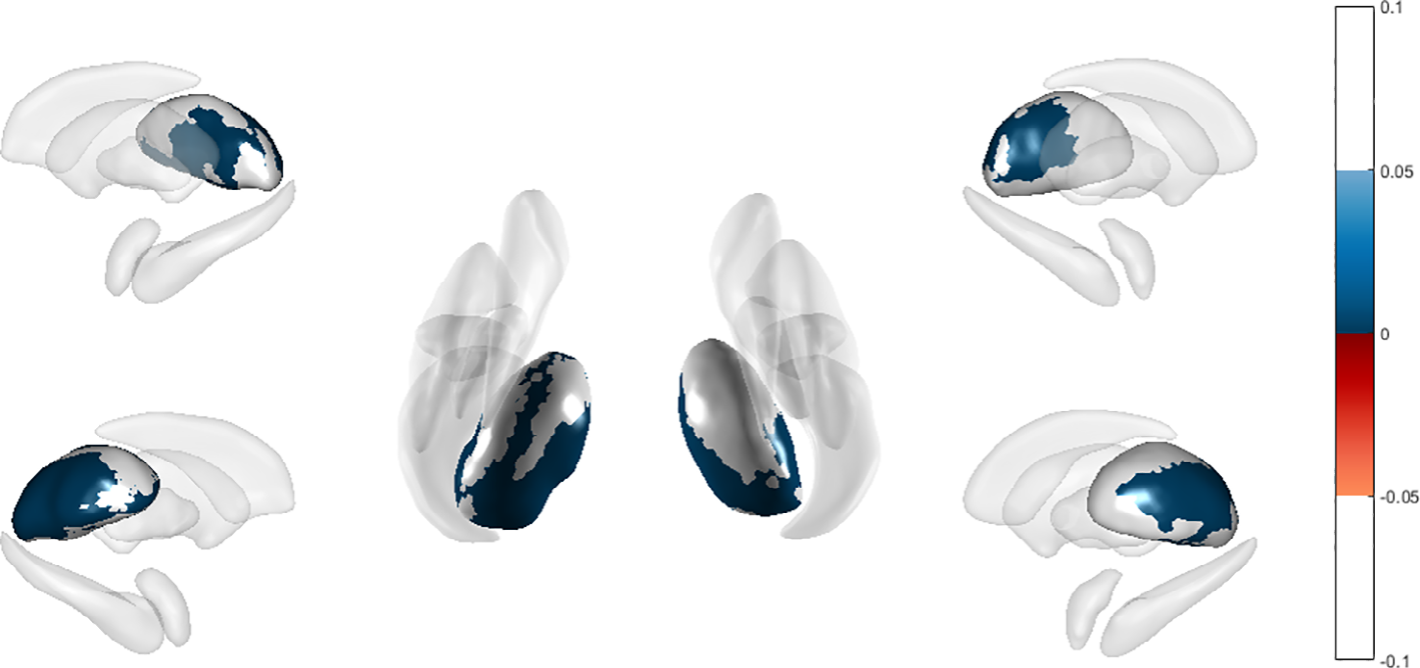
Subcortical structures associated with the duration of illness in the suicidal major depressive disorder group. Positive correlations are *blue*, and negative correlations are *red*. Bilateral thalamus had positive correlation with the duration of illness in the suicidal major depressive disorder group. Color bar shows adjusted *p* value (cluster‐based statistics method)

In the SA group, both thalamic regions demonstrated a positive correlation with the durations of illness (Figure 2) and antidepressant use. On the other hand, they had a negative correlation with SSI (Table 3). The duration of medication treatment was negatively correlated with the left amygdala, left hippocampus, and left putamen. Both the putamen and pallidum were found to be positively correlated with the PSS (Table 3).

**TABLE 3:** Correlations between the clinical scale and LSV of SA and NS

Group	Clinical scales	Subcortical structures	*t*	Cluster	Size	*p*
SA	Duration of antidepressant	L amygdala	−0.3	1	369	.0082
				2	269	.017
		L hippocampus	−0.3	1	164	.046
		L putamen	−0.3	1	444	.0202
		L thalamus	0.3	1	284	.0328
		R thalamus	0.3	1	344	.0288
	Duration of illness	L thalamus	0.3	1	1,696	.0026
		R thalamus	0.3	1	1,058	.0046
	SSI	L thalamus	−0.3	1	197	.0426
		R thalamus	−0.3	1	419	.0186
	PSS	L pallidum	0.3	1	164	.0346
		L putamen	0.3	1	209	.0424
		R pallidum	0.3	1	153	.0444
		R putamen	0.3	1	491	.0112
NS	HDRS‐17	L amygdala	0.3	1	44	.0064
				2	16	.0318
		R hippocampus	−0.3	1	35	.0126
				2	19	.0266
		R pallidum	0.3	1	10	.0304

Abbreviations: BIS, Baratt Impulsiveness Scale; corrected *p*, FDR adjusted *p*‐value; FDR, false discovery rate; HC, healthy control; HDRS, Hamilton Depression Rating Scale; LSV, local shape volume; MDD, major depressive disorder; NS, nonsuicidal MDD; PSS, Perceived Stress Scale; SA, suicide attempted MDD; SSI, Beck Scale for Suicide Ideation.

## 4 DISCUSSION

The major finding of this study was that the SA group had significant LSV alterations in the left putamen, left hippocampus, left amygdala, bilateral pallidum, and thalamus. Our findings are in line with the subcortical structural changes in suicidal depression (Ahearn et al., 2001; Monkul et al., 2007; Vang et al., 2010), despite studies with contradictory results (Gifuni et al., 2016; Jollant et al., 2018; Kim et al., 2019; Rentería et al., 2017). Compared with the NS group, the SA group showed significantly higher SSI, longer illness duration, and an increased number of past episodes. However, these two groups were not significantly different in the BIS, which indicate that the SA group had more suicidal ideations and an intent to die, rather than just impulsively attempting suicide. The clinical data showed that the SA group entertained suicidal ideation along with a long illness duration and high stress levels, and these conditions were associated with structural changes of the basal ganglia and thalamus. However, we found no significant differences between the NS and HC groups in regions other than the right amygdala, hippocampus, caudate, and thalamus. Among these regions, the right hippocampus showed the largest LSV changes (Table 2), which correlated with the severity of depression (Table 3). Imaging studies on the subcortical volumes in patients with depression and their relationship with depression severity have reported inconsistent results, but the hippocampal volumetric decrease has been a consistent finding (Chen et al., 2020; Schmaal et al., 2016; Zhao et al., 2017).

Our findings of LSV alterations suggest the disruption of subcortical regions among the limbic–cortico–striatal–pallidal–thalamic (LCSPT) circuits, especially on the left side of the brain, in patients with SA. The LCSPT circuits are critical neuroanatomic pathways that connect subregions of the prefrontal cortex and the anterior cingulate cortex with the basal ganglia and thalamus in an organized and integrated manner to support diverse motor, cognitive, and emotional processes (Alexander, DeLong, & Strick, 1986; Haber & Calzavara, 2009; Swerdlow & Koob, 1987). Early imaging studies found that disruptions of these regions were associated with vulnerability to affective dysfunction (Koshiyama et al., 2018; Krishnan, 1991). This concept was adopted because the dysfunction and main pathological symptoms were too diverse to be explained by a single lesion (Sheline, 2003). Neuroimaging studies have provided supportive evidence that structural deficits of brain networks regulating affective evaluation are involved in the pathophysiology of mood disorders (Phillips, Drevets, Rauch, & Lane, 2003; Price & Drevets, 2010). This circuitry approach in underpinning suicidality in depression has been supported by several other recent studies (Gosnell et al., 2016; Jia et al., 2014; Peng, Chen, Yin, Jia, & Gong, 2016; Zhang et al., 2014).

The altered LSV of the subcortical structures over the left LCSPT circuits in the SA group may be associated with LSV changes in the putamen and pallidum and their relationship with stress. Although the LSV atrophies were found only in the left side of putamen and pallidum, the positive correlations with stress level were found in both sides. The alterations in one region might be followed by a subsequent volume change in the connected regions, influencing the entire network as a consequence (Kim, Hamilton, & Gotlib, 2008). This may explain how the SA group showed the left‐sided atrophies through the LCSPT circuits. The understanding of suicidality is based on a stress‐diathesis model, which states that suicidal ideation is triggered by an interplay between state‐dependent factors (i.e., psychosocial crisis) and vulnerability factors (e.g., MDD) (Mann, 2003). If the PSS is more state dependent, the BIS indicates a trait of the person. There was no significant difference in PSS and BIS between the SA and NS group. Based on our demographic information, the SAs attempted suicide an average of 1.64 times with a maximum number of 7. Some studies proposed that suicidal behavior was associated with impulsivity (Kim et al., 2019). However, we found no significant correlation between the BIS and subcortical LSV changes. Thus, in our results, the stress may better explain suicidal behavior than impulsivity does. Over the significantly longer duration of illness, the SA group may have experienced repeated exposure to stressful life events. Subsequently, dwindling resilience toward the same type of stressors may lead to be more vulnerable to suicidal ideations and behaviors (Nye et al., 2013).

The novel finding of the current study is that the thalamus is the most sensitive subcortical structure in the SA group. The thalamic volume was found to be correlated positively with the durations of illness and antidepressant usage, and negatively correlated with SSI in the SA group. Previous studies reported the thalamic volume changes in mood disorders (Bora, Harrison, Davey, Yücel, & Pantelis, 2012; Young, Holcomb, Yazdani, Hicks, & German, 2004) and those with suicidal behavior (Lopez‐Larson et al., 2013). In particular, a postmortem study (Young, Bonkale, Holcomb, Hicks, & German, 2008) reported that patients with psychiatric disorders who died from suicide had a thalamus enlarged by 8%, while antidepressant treatment was associated with an 18% lower total thalamic volume. Our results add to the evidence of changes in thalamic volume with SA. However, the association with antidepressants was contrary to previous study results. The thalamus is the last stop of the LCSPT circuits, acting as a bridge between different subcortical and cortical structures, especially for the inhibitory pons (Wakana, Jiang, Nagae‐Poetscher, van Zijl, & Mori, 2004). Given that the structural changes indicate that the functions of the region are altered over time (Bielau et al., 2005), the thalamic volume could increase to compensate for the increased disease burden as the illness progresses. Other possible explanations could be an effect of antidepressant treatment or it could be a combination of both disease burden and treatment effects.

Although the recent findings failed to detect significant volumetric changes of the subcortical structures in MDD patients with a history of suicide attempts, we found LSV changes in many subcortical structures with clinical correlations. The inconsistent findings of previous studies are often attributed to the sample size, resulting from the difficulties in collecting data of those with suicidal depression, the heterogeneity of the samples such as antidepressant treatment, and the different methodology of each study.

Our study also had some limitations in this regard. First, it was conducted with a limited sample size and low power. The power analysis, conducted based on the total volume of the subcortical structure, indicated that given the observed Cohen's *d* values, the present study requires larger samples for both the SA and NS groups (*N* = 112; power level = 0.8; *p* = .05; two‐tailed test). Second, patients with MDD had heterogeneous concomitant medications and durations of illness. As for the significantly longer illness duration in the SA group, most SA patients were patients on follow‐up, whereas more than half of the NS patients were new patients and were antidepressant drug‐naïve. This may have affected the results as a confounding factor. The present study also adopted a vertex‐wise shape analysis, a relatively new approach, compared with the conventional voxel‐based approach that can detect more subtle changes in the structures. Despite its sensitivity, this new method has yet to be sufficiently replicated. There were also minor limitations such as the detailed condition of suicidal attempters not being taken into account. For example, suicide attempts that were carefully planned over a long time and impulsive suicidal attempts may have different etiologies (Witte et al., 2008). Also, the present study provided structural changes in limbic, striatal, pallidal, and thalamic volumes but not the cortical volumes. To better clarify the relationship between the LCSPT circuits and suicidality in depression, future studies should examine the cortical and subcortical regions together.

In summary, we demonstrated that subcortical structures were significantly reduced in LSV in depressed patients with a history of suicide attempt. Our findings support the stress‐diathesis model (Mann, 2003), explaining suicidal behavior by vulnerability due to MDD and the personal state of mood with stressful life events. Our results demonstrated that the basal ganglia correlated with the perceived stress levels while that the thalamus correlated with suicidal ideation. These findings propose that suicidality in MDD may involve subcortical volumetric alterations.

## CONFLICT OF INTEREST

The authors declare no conflict of interest.

## Supporting information


**Supplementary Figure S1** LSV differences between SA and NS controlling for the effects of age, sex, level of education, intracranial volume, SSI score, and antidepressant treatment duration. HC, healthy control; MDD, major depressive disorder; SA, suicide attempted MDD; NS, non‐suicidal MDD; SSI, Beck Scale for Suicide Ideation.

**Supplementary Table S1** Correlations between the clinical scale and LSV of SA and NS

## Data Availability

The data that support the findings of this study are available from the corresponding author upon reasonable request.
