# Supplementary figures and images for: Local shape volume alterations in subcortical structures of suicide attempters with major depressive disorder
Source: Hum Brain Mapp. 2020 Aug 17;41(17):4925–34. doi: 10.1002/hbm.25168 (PMC7643352; doi:10.1002/hbm.25168)

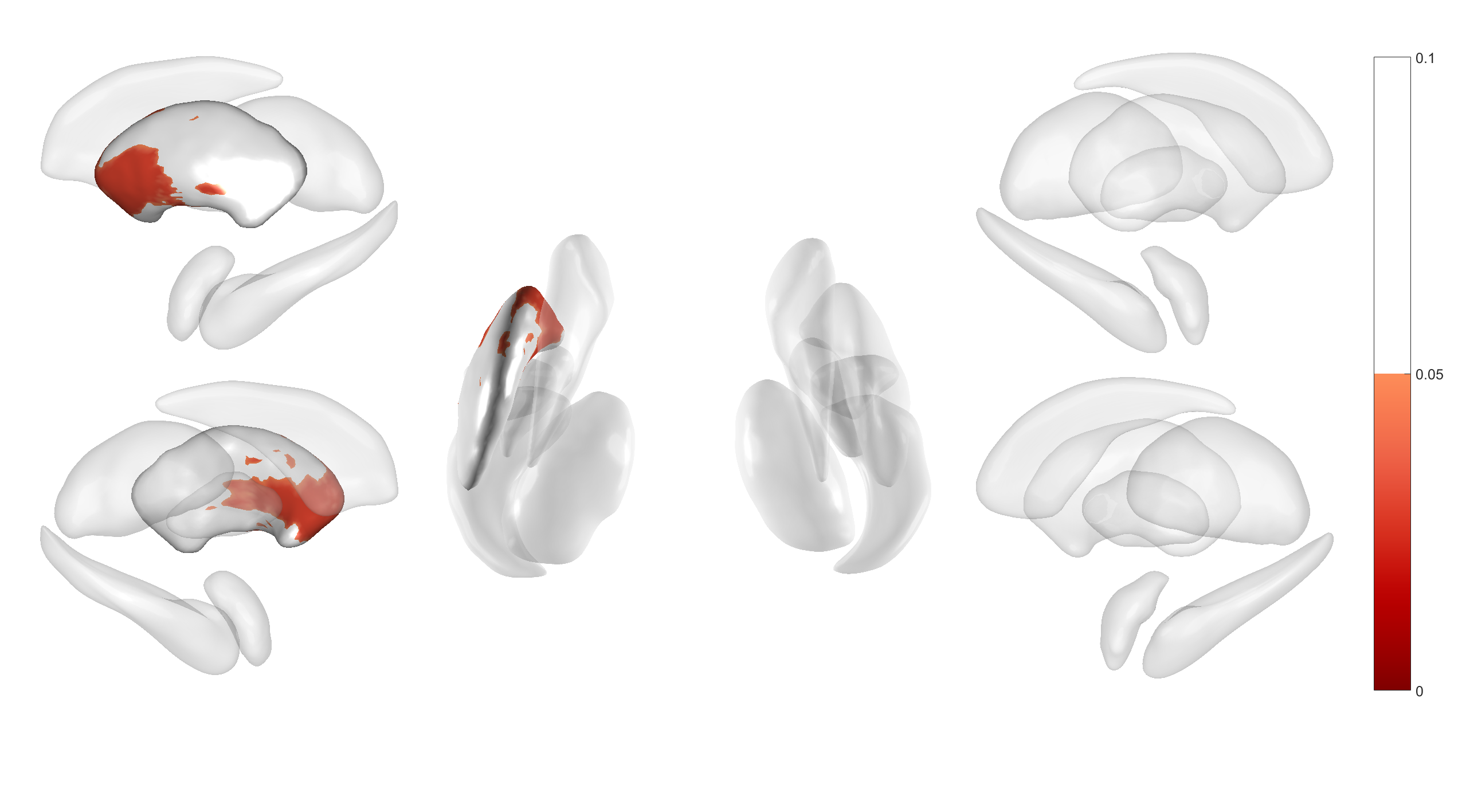

Supplement: Supplementary file 1 — Supplementary Figure S1 LSV differences between SA and NS controlling for the effects of age, sex, level of education, intracranial volume, SSI score, and antidepressant treatment duration. HC, healthy control; MDD, major depressive disorder; SA, suicide attempted MDD; NS, non‐suicidal MDD; SSI, Beck Scale for Suicide Ideation. [file HBM-41-4925-s001.tif]
