# Supplementary material for: Local shape volume alterations in subcortical structures of suicide attempters with major depressive disorder
Source: Hum Brain Mapp. 2020 Aug 17;41(17):4925–34. doi: 10.1002/hbm.25168 (PMC7643352; doi:10.1002/hbm.25168)
Supplement: Supplementary file 2 — Supplementary Table S1 Correlations between the clinical scale and LSV of SA and NS [file HBM-41-4925-s002.docx]

**Supplementary Table 1.** Correlations between the clinical scale and LSV of SA and NS

|  |  | **NS** | | **SA** | |  | **NS** | | **SA** | |
| --- | --- | --- | --- | --- | --- | --- | --- | --- | --- | --- |
| Subcortical structures | Clinical scales | pos | neg | pos | neg | Clinical scales | pos | neg | pos | neg |
| L Amygdala | **HDRS-17** | 63 | 0 | 6 | 49 | **SSI** |  |  | 0 | 155 |
| L Caudate |  | 0 | 6 | 34 | 0 |  |  |  | 30 | 1 |
| L Hippocampus |  | 0 | 1 | 37 | 21 |  |  |  | 1 | 0 |
| L Pallidum |  | 0 | 0 | 50 | 0 |  |  |  | 4 | 0 |
| L Putamen |  | 0 | 0 | 10 | 0 |  |  |  | 7 | 2 |
| L Thalamus |  | 0 | 0 | 0 | 166 |  |  |  | 0 | 394 |
| R Amygdala |  | 4 | 0 | 0 | 26 |  |  |  | 5 | 215 |
| R Caudate |  | 0 | 0 | 30 | 0 |  |  |  | 49 | 5 |
| R Hippocampus |  | 8 | 60 | 246 | 0 |  |  |  | 44 | 0 |
| R Pallidum |  | 10 | 0 | 3 | 0 |  |  |  | 4 | 64 |
| R Putamen |  | 0 | 0 | 3 | 0 |  |  |  | 21 | 1 |
| R Thalamus |  | 0 | 0 | 0 | 154 |  |  |  | 0 | 520 |
| L Amygdala | Duration of antidepressant |  |  | 33 | 677 | **BIS** |  |  | 59 | 60 |
| L Caudate |  |  |  | 3 | 1 |  |  |  | 17 | 0 |
| L Hippocampus |  |  |  | 0 | 190 |  |  |  | 17 | 33 |
| L Pallidum |  |  |  | 13 | 80 |  |  |  | 0 | 202 |
| L Putamen |  |  |  | 0 | 448 |  |  |  | 205 | 0 |
| L Thalamus |  |  |  | 339 | 0 |  |  |  | 7 | 44 |
| R Amygdala |  |  |  | 53 | 1 |  |  |  | 22 | 82 |
| R Caudate |  |  |  | 2 | 55 |  |  |  | 3 | 60 |
| R Hippocampus |  |  |  | 0 | 214 |  |  |  | 0 | 65 |
| R Pallidum |  |  |  | 1 | 33 |  |  |  | 2 | 29 |
| R Putamen |  |  |  | 1 | 104 |  |  |  | 47 | 0 |
| R Thalamus |  |  |  | 466 | 0 |  |  |  | 0 | 62 |
| L Amygdala | **Duration of illness** |  |  | 28 | 88 | **PSS** |  |  | 38 | 152 |
| L Caudate |  |  |  | 26 | 9 |  |  |  | 110 | 0 |
| L Hippocampus |  |  |  | 38 | 13 |  |  |  | 2 | 0 |
| L Pallidum |  |  |  | 176 | 0 |  |  |  | 315 | 7 |
| L Putamen |  |  |  | 30 | 11 |  |  |  | 328 | 0 |
| L Thalamus |  |  |  | 1703 | 0 |  |  |  | 1 | 29 |
| R Amygdala |  |  |  | 106 | 0 |  |  |  | 6 | 130 |
| R Caudate |  |  |  | 304 | 3 |  |  |  | 171 | 9 |
| R Hippocampus |  |  |  | 2 | 25 |  |  |  | 123 | 5 |
| R Pallidum |  |  |  | 171 | 4 |  |  |  | 224 | 22 |
| R Putamen |  |  |  | 89 | 3 |  |  |  | 525 | 15 |
| R Thalamus |  |  |  | 1144 | 0 |  |  |  | 11 | 6 |

HC, healthy control; MDD, major depressive disorder; SA, suicide attempted MDD; NS, non-suicidal MDD; pos, the number of vertices with correlation coefficient >0.3 and uncorrected p-value < 0.05; neg, the number of vertices with correlation coefficient <-0.3 and uncorrected p-value < 0.05
